# Novel 3D-navigated screw corridors for posterior pelvic ring stabilization: a finite element analysis

**DOI:** 10.1007/s00068-025-03053-9

**Published:** 2026-01-13

**Authors:** Eftychios Bolierakis, Maximilian Praster, Hatem Alabdulrahman, Ulf Krister Hofmann, Christian Herren, Roman Michalik, Michel Teuben, Hans-Christoph Pape, Frank Hildebrand, Till Berk

**Affiliations:** 1 Department of Orthopaedics, Trauma and Reconstructive Surgery, Medical Faculty, RWTH Aachen University, Pauwelsstraße 30, 52074 Aachen, Germany; 2 Department of Orthopaedics, Trauma and Reconstructive Surgery, Division of Arthroplasty, Medical Faculty, RWTH Aachen University, Pauwelsstraße 30, 52074 Aachen, Germany; 3 Department of Trauma, University Hospital Zurich, Raemistrasse 100, Zurich, 8091 Switzerland; 4 Harald-Tscherne Laboratory for orthopedic and Trauma research, University of Zurich, Sternwartstrasse 14, Zurich, 8091 Switzerland; 5 Department of Orthopedics, Trauma and Reconstructive Surgery, RWTH Aachen University, Aachen, Germany

**Keywords:** Posterior pelvic ring disruption, Finite element model, Novel sacrum osteosynthesis, 3 d navigation, Orthopedics, Screw corridors

## Abstract

**Purpose:**

Posterior pelvic ring stabilization is technically demanding due to the complex local anatomy and limited osseous corridors. Advances in 3D navigation may allow for new screw trajectories previously infeasible with conventional fluoroscopy.

**Methods:**

A finite element model (FE) of a pelvis (Synbone LSS4060/Hard^®^) was developed, simulating seven screw configurations, including three novel oblique navigated pathways (Variants III–V). A 600 N vertical load was applied to simulate bipedal stance. Stress distribution and von Mises stresses were compared among configurations.

**Results:**

Conventional transsacral screws (Variants I–II) demonstrated the most uniform stress distribution and lowest peak stresses (7.17 MPa). Among novel configurations, Variant III achieved favorable compressive load transfer and lower shear stresses. Variant IV exhibited the highest overall stress (17.80 MPa), while Variant V demonstrated intermediate behavior.

**Conclusion:**

The proposed 3D-navigated oblique screw pathways are anatomically feasible and may offer biomechanical advantages under certain conditions. These findings support further validation using cadaveric and clinical studies.

## Introduction

Pelvic injuries present a challenge for orthopedic trauma surgeons due to the complex pelvic anatomy and the need for stable fixation through narrow osseous corridors located near vital neurovascular structures. The posterior pelvic ring plays a fundamental role in maintaining the structural integrity and function of the pelvis. Accurate and rapid screw placement in this anatomical region is essential for restoring its load-bearing capacity, enabling fast and early rehabilitation, and preventing complications such as neurovascular injury, infections, or implant failure [1].

Traditionally, screw fixation of posterior pelvic ring fractures is achieved in a minimally invasive manner under fluoroscopic imaging control to guide appropriate intraosseous screw placement. In many cases, this is performed through the iliac bone combined with the first and/or second sacral vertebra. However, in certain circumstances, successful performance of the operation under conventional fluoroscopy alone can be very challenging, especially in displaced multifragmentary fractures or when visualization of anatomical structures is limited due to osteoporotic bone quality or dysplastic sacral anatomy. Also overprojection of intestinal gases can hinder appropriate visualization of the posterior pelvic ring. All these aspects may compromise precise screw placement [2]. The emergence of navigation-assisted osteosynthesis, particularly with the implementation of computer-assisted and CT-based navigation protocols, has greatly enhanced the accuracy and safety of screw placement in the pelvic ring [3].

Navigation systems use preoperatively acquired imaging data to create a virtual model of the patient’s anatomical osseous structures, such as the pelvis. This allows the surgeon to visualize the osseous target corridors, and to plan and perform accurate screw placement according to the preoperative plan. This technology can thus ensure an optimal screw trajectory and minimize risks associated with misplacement, such as injury to surrounding neurovascular structures or compromised fracture stability [4]. Hence, this technology contributes to better clinical outcomes and lower reoperation rates [3]. Additionally, the utilization of navigation leads to reduced intraoperative fluoroscopy time and, thus, less radiation exposure for the patient and operating room staff [3, 5]. With further technological advances and navigation systems becoming increasingly integrated into routine practice, new opportunities are created for alternative and possibly more stable types of fixation.

Aim of this finite element (FE) study was to evaluate the feasibility of novel 3D-navigated screw corridors for the posterior pelvic ring and evaluate their principal biomechanical properties on the theoretical FE model. We hypothesized, that alternative, previously not exploited, osseous screw corridors can be feasible and potentially biomechanically advantageous.

## Materials and methods

### Specimens and SI-joint dislocation model

In this study, a Finite Element Analysis (FEA) was performed using the Abaqus software (Version 2019, Dassault Systèmes, Vélizy-Villacoublay, France) to analyze the system, while the geometry was generated using SpaceClaim (Ansys, Inc., Canonsburg, Pennsylvania, USA). The geometry of the pelvis was derived from an orthopaedic model manufactured by Synbone [6]. The geometry in question delineates a male pelvis with a width of 305 mm, a height of 160 mm, and an acetabular diameter of 56 mm. The synthetic pelvic model (model LSS4060/Hard^®^, Synbone, Zizers, Switzerland) was scanned by CT, digitized to an STL-file and then transformed into a volume model [7, 8]. In the next step, the screws were modeled as smooth cylindrical rods (6.5 mm) without threads and bonded to the bone surface. Although the average screw diameter implanted in the male pelvis is 7–8 mm, the 6.5 mm screw diameter was chosen for study purposes to facilitate generalizability of the study results on female and/or dysmorphic pelvis, where the transsacral osseous corridors, if existent, are narrower [9, 10]. Two curved surfaces were modeled with a distance of up to 2 mm to create a SI-joint gap in the pelvic geometry, as described in the literature [11]. A posterior SI-joint dislocation injury type III B according to the fragility fractures of the pelvis (FFP) classification was simulated on all pelvic models [12]. For better visualization of the placement of the different screws in the test variants combined with the screw angles in the frontal plane (α), transverse plane (β) and sagittal plane (γ) (Table 1). In the global coordinate system (XYZ), the Z-axis represents the vertical direction, the Y-axis the medial-lateral axis, and the X-axis the anterior-posterior axis. The angles α, β, and γ describe rotations in the frontal, transversal, and sagittal planes, respectively, with α corresponding to rotation around the X-axis, β around the Z-axis, and γ around the Y-axis.

**Table 1:** Lengths and position angles of the test variants I-VII

Study Variant	Screw Length (mm)	α (°)	β (°)	γ (°)
Variant I	184.0	0	0	0
Variant II	184.0/157.0	0	0	0
Variant III	155.3	83.2	−2.0	−74.2
Variant IV	130.9	−53.9	14.3	−70.8
Variant V	162.1	72.7	−7.6	−66.9
Variant VI	129.4	−24.1	11.0	−85
Variant VII	129.4/100.0	−24.1/−33.7	11.0/6.0	−85/−86.0

### Study groups

The pelvis models were stratified into seven groups for simulated instrumentation as follows (Appendix 1: Fig. 7, 8, 9, 10, 11, 12 and 13)

- Variant I: one horizontal transiliac transsacral screw in the S1 osseous sacral corridor through both S1 pedicles (diameter: 6.5 mm, length: 184.0 mm).
- Variant II: two horizontal transiliac transsacral screws, one screw in in the S1 osseous sacral corridor through both S1 pedicles (diameter: 6.5 mm, length: 184.0 mm) and one screw in the S2 osseous sacral corridor through both S2 pedicles (diameter: 6.5 mm, length: 157.0 mm).
- Variant III: one oblique sacral screw through the right S1 pedicle to the contralateral sacral ala at the S2 level (diameter: 6.5 mm, length: 155,3 mm).
- Variant IV: one oblique sacral screw through the right S2 pedicle to the contralateral sacral ala at the S1 level (diameter: 6.5 mm, length: 130,9 mm).
- Variant V: one oblique sacral screw through the right S1 pedicle to the contralateral sacral cortical margin at the S3 level (diameter: 6.5 mm, length: 162,1 mm).
- Variant VI: one horizontal sacral screw through the right S1 pedicle to the contralateral sacral ala at the S1 level (diameter: 6.5 mm, length: 129,4 mm).
- Variant VII: two horizontal sacral screws, one screw in the S1 osseous sacral corridor through the right S1 pedicle to the contralateral sacral ala (diameter: 6.5 mm, length: 129,4 mm) and one screw in the S2 osseous sacral corridor through the right S2 pedicle to the contralateral sacral ala (diameter: 6.5 mm, length: 100.0 mm).

### Novel osseous screw pathways

Variants III to V are novel suggested pathways through the posterior pelvic ring:

- In Variant III, the screw enters the ilium through a conventional starting point at the S1 level, transverses the sacrum obliquely centrally at level of S1-S2 osseous junction and ends at the contralateral sacral ala at the S2 level [13].
- In Variant IV, the screw enters the ilium through a conventional starting point at the S2 level, transverses the sacrum obliquely centrally at level of S1-S2 osseous junction and ends at the contralateral sacral ala at the S1 level [13].
- In Variant V, the screw enters the ilium through a conventional starting point at the S1 level, transverses the sacrum obliquely centrally at level of the S2 vertebra and ends at the lateral cortex at the S3 level [13].

### Data acquisition & analysis

**Fig. 1:**
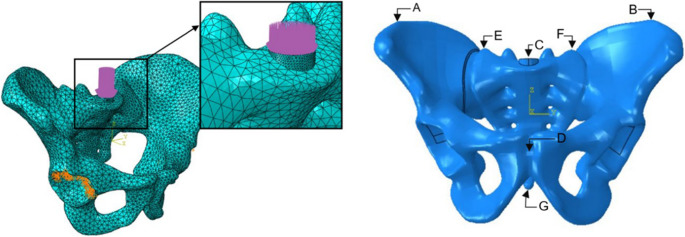
Left: Support and load situation for the seven variants to be simulated Right: Representation of the pelvis with significant examination points

The model of the cylindrical load base (*r* = 10 mm) of the FE models enables the pressure application to a predefined and horizontally aligned surface so that there are no fluctuations in the load height and direction between the test variants. A vertical load of 600 N was added to the load base in the form of surface pressure. The load level was selected based on existing FE studies (Fig. 1 Left) [14].

For the FE analysis, the sacroiliac (SI) screws were placed by a senior pelvic trauma surgeon and independently validated by another independent senior pelvic trauma surgeon.

A static bipedal stance was chosen for loading, and the supports in the acetabulum were assumed to be rigid. The simulated injury occured at point E (Fig. 1 Right).

The pelvic structures were meshed using square tetrahedral elements, and these meshes were refined until convergence was achieved (Table 2). A linear elastic material model was assumed for both the bone and screws to simplify the analysis of their biomechanical behavior under applied loads (Table 3) [15].

**Table 2:** Number of elements and form factor of test variants I-VII

Study Variant	Number of Elements	Form factor
Variant I	57,411	0.628594
Variant II	69,762	0.614952
Variant III	68,424	0.628424
Variant IV	65,526	0.631342
Variant V	79,667	0.620384
Variant VI	76,351	0.618291
Variant VII	85,894	0.618100

**Table 3:** Material properties used in the finite element model

Component	Young’s modulus [GPa]	Poisson Ratio
Pelvic	1.835	0.3
Screws	110	0.3

**Fig. 2:**
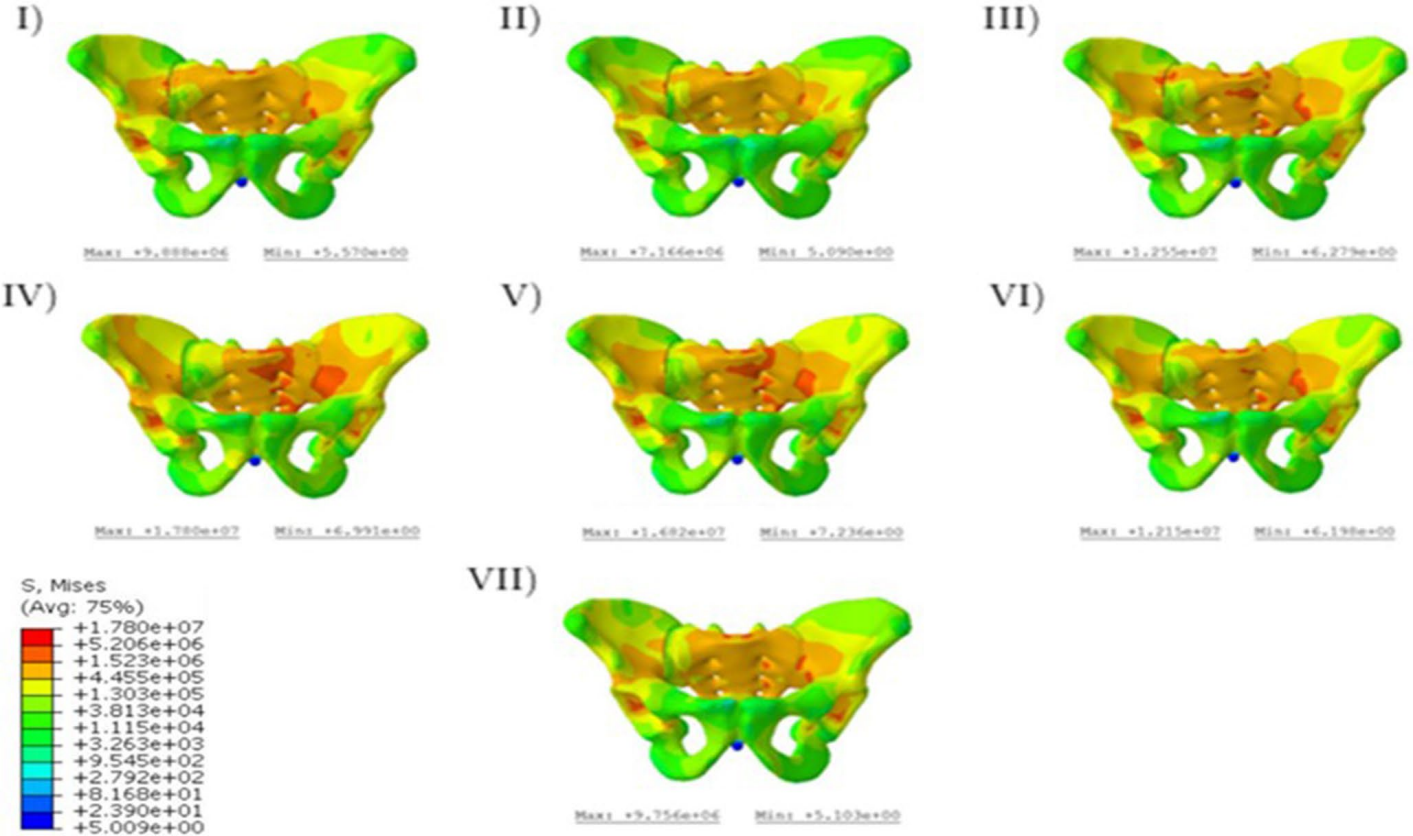
Von Mises stresses in the pelvic bones

The stress curves resulting from the vertical load can be observed in Fig. 2. The von Mises stress was used as a reference value [16]. To enable a quantitative comparison in the graphical representation, a uniform legend was chosen for all variants. The course of the forces begins in all variants at the load application point, the sacrum, then leads via the sacroiliac joint. For all variants, the force curve begins at the load application point, the sacrum, and then runs via the sacroiliac joint along the linea terminalis to the supports.

## Results

A particularly uniform stress distribution is achieved with the transsacral screws (I, II), while.

the sacroiliac screws (III-VII) show a significantly higher stress distribution along the non-dislocated side. In these variants, high stress areas are evident along the linea terminalis and between the anterior sacral foramina. The new osteosynthesis variants with iliosacral screws (III-V) show higher unilateral stress compared to the conventional ones (VI, VII). The stress distribution exhibits a notably uniform pattern, with a regular gradient indicated by color mapping, suggesting a consistent variation in stress across the material. In variants III-V, the maximum stress values are observed to reach up to 15 MPa, which is 1.5 times greater than the maximum stress of 9.8 MPa recorded in the conventional variants. In addition, the first and third variants show significant increases in stress in the area of the simulated gap. Overall, the maximum stresses are the highest in the fourth variant at 17.80 MPa, while they are the lowest in Variant II at 7.17 MPa (Fig. 2).

**Fig. 3:**
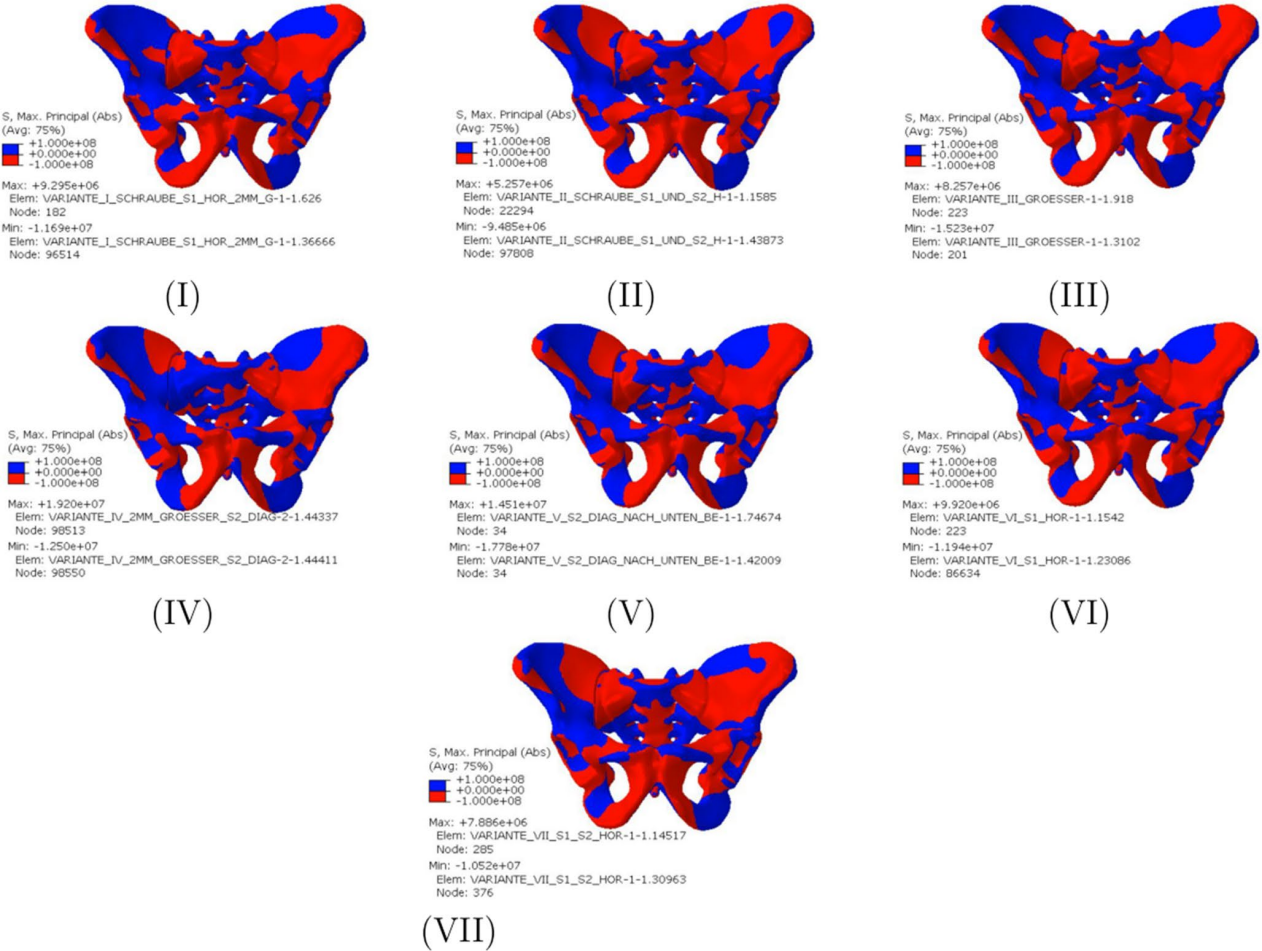
Representation of the highest principal stress in the pelvic bone in terms of magnitude

Figure 3 illustrates the highest principal stresses in terms of magnitude on the pelvic bones. For illustration, differentiation between negative and positive values was chosen so that a statement can be made about the areas in which tensile or compressive stresses occur. Overall, a concentration of compressive stress on the undisturbed side can be seen in all variants. The compressive stresses run along a circular or loop shape along the linea terminalis and the iliac crest. Variants II, IV, V, VI and VII are subjected to pressure in the dorsal area, while Variants I and III are subjected to pressure on the inside of the sacroiliac joint in the direction of the linea terminalis. When evaluating the sacrum, it can be noticed that the pressure and tension distribution is particularly symmetrical among the variants with two horizontal screws (II, VII). The highest pressure and tension stresses can be assigned to the new Variants IV and V. The new Variant III shows similar values to the conventional sacroiliac screws (VI, VII). Only the pressure stress of the Variant III is higher at 15.23 MPa than that of a conventional sacroiliac screw (VI). The lowest stresses can be attributed to the first two variants.

**Fig. 4:**
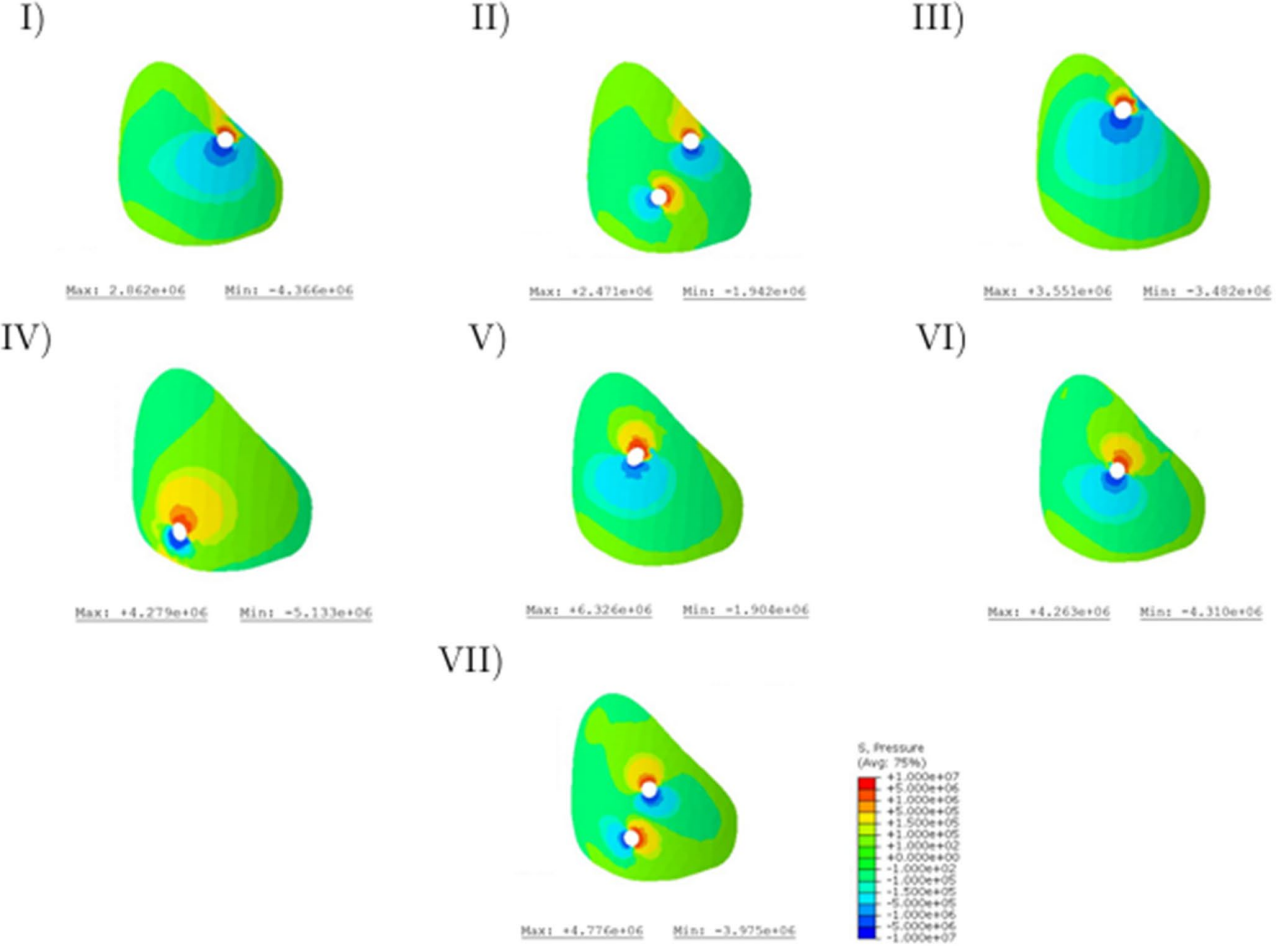
Von Mises stresses of the left gap surface

To illustrate the effects on injury retention, Fig. 4 presents the von Mises stresses on the left gap surface. The graph shows that the stresses in the area surrounding the screw entry hole are increased in all variants. Especially Variant IV is characterized by a large area with increased stresses. The enhanced stress appears to be low for the second screws in Variants II and VII. Furthermore, a consistent increase in stress toward the dorsal aspect of the pelvis was observed, indicating higher load concentrations near the sacral region. However, a more constant stress curve can be observed in Variant III. The numerical comparison of the maximum stresses shows that they occur in the following order: V; VII, IV; VI; III; I; II (Fig. 4). In further comparison, the ratio between the maximum and minimum stresses was calculated (Table 4).

**Table 4:** Minimum and maximum values on the gap surface for the different variants

Variant	Hydrostatic pressure [MPa] (Min/Max)	Principal strain (Min/Max)
I	−4.37/2,86	−0.31/0.45
II	−1.94/2.47	−0.28/0.20
III	−3.48/3.55	−0.53/0.49
IV	−5.13/4.28	−0.71/0.68
V	−1.90/6.33	−0.97/0.92
VI	−4.31/4.26	−0.60/0.51
VII	−3.98/4.78	−0.48/0.39

**Fig. 5:**
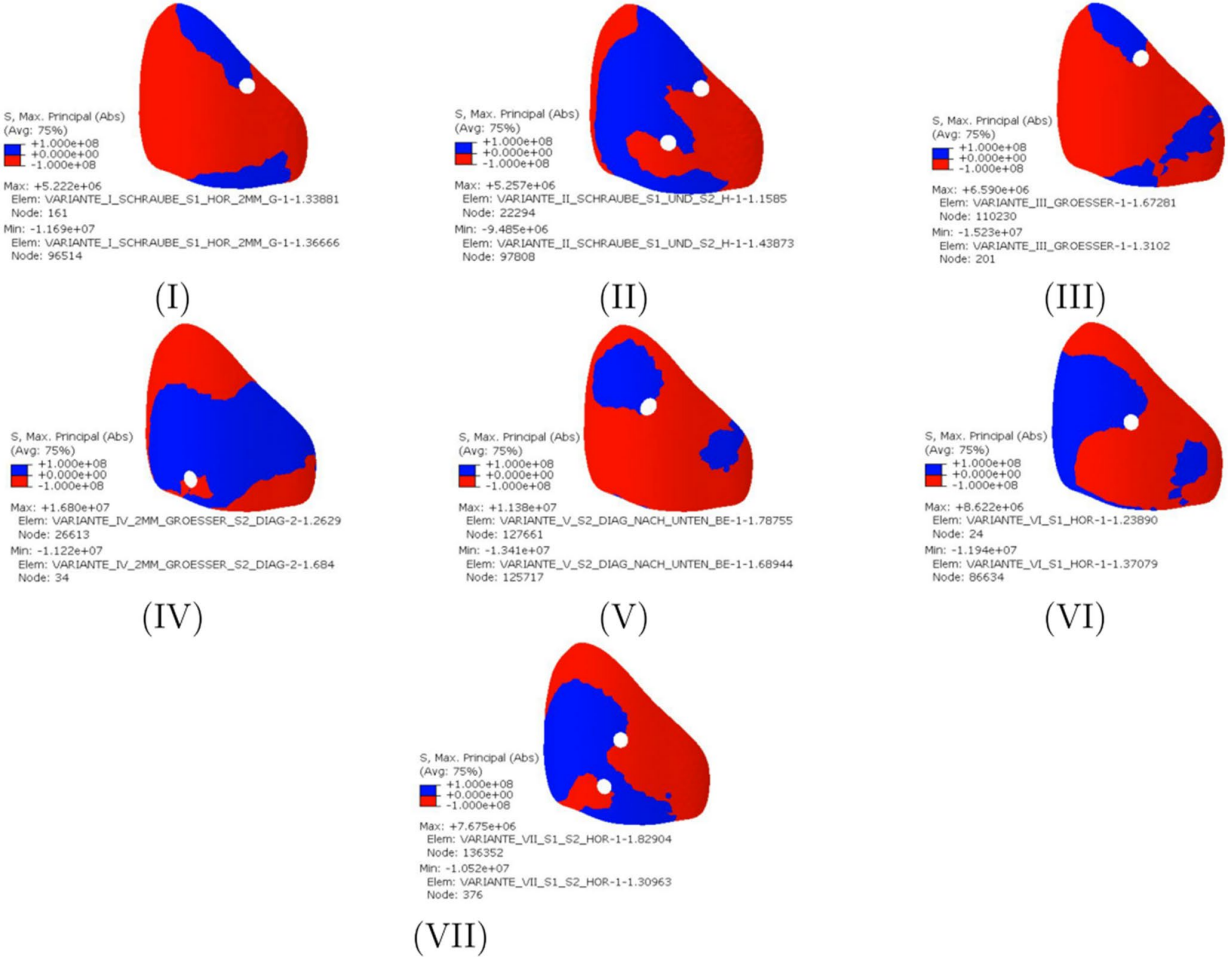
Maximum principal stresses on the left gap surface

In order to minimize the influence of singularities in the maximum range, the same comparison was also carried out with a mean stress (set at 0.22 MPa). This comparison showed that the ratio is particularly favorable in the third variant, while in the conventional variants with sacroiliac screws (VI; VII) it leads to unfavorably high values (Fig. 5).

High compressive stress areas can be seen in the conventional Variants I and VI. When two screws are used, the compressive stress area is reduced (Variants II and VII). Similar high compressive stress areas can be seen in the new Variants III and V. The highest difference between compressive and tensile stresses can be observed in Variant IV. In addition, the highest tensile stresses of 16.80 MPa are also observed in screw position Variant IV.

The smallest difference between compressive and tensile stress occurs when using.

two conventional transsacral screws (variant II). In Variant III, the differences between compressive and tensile stress are comparable to those in conventional iliosacral screw fixation (Variant VI).

**Fig. 6:**
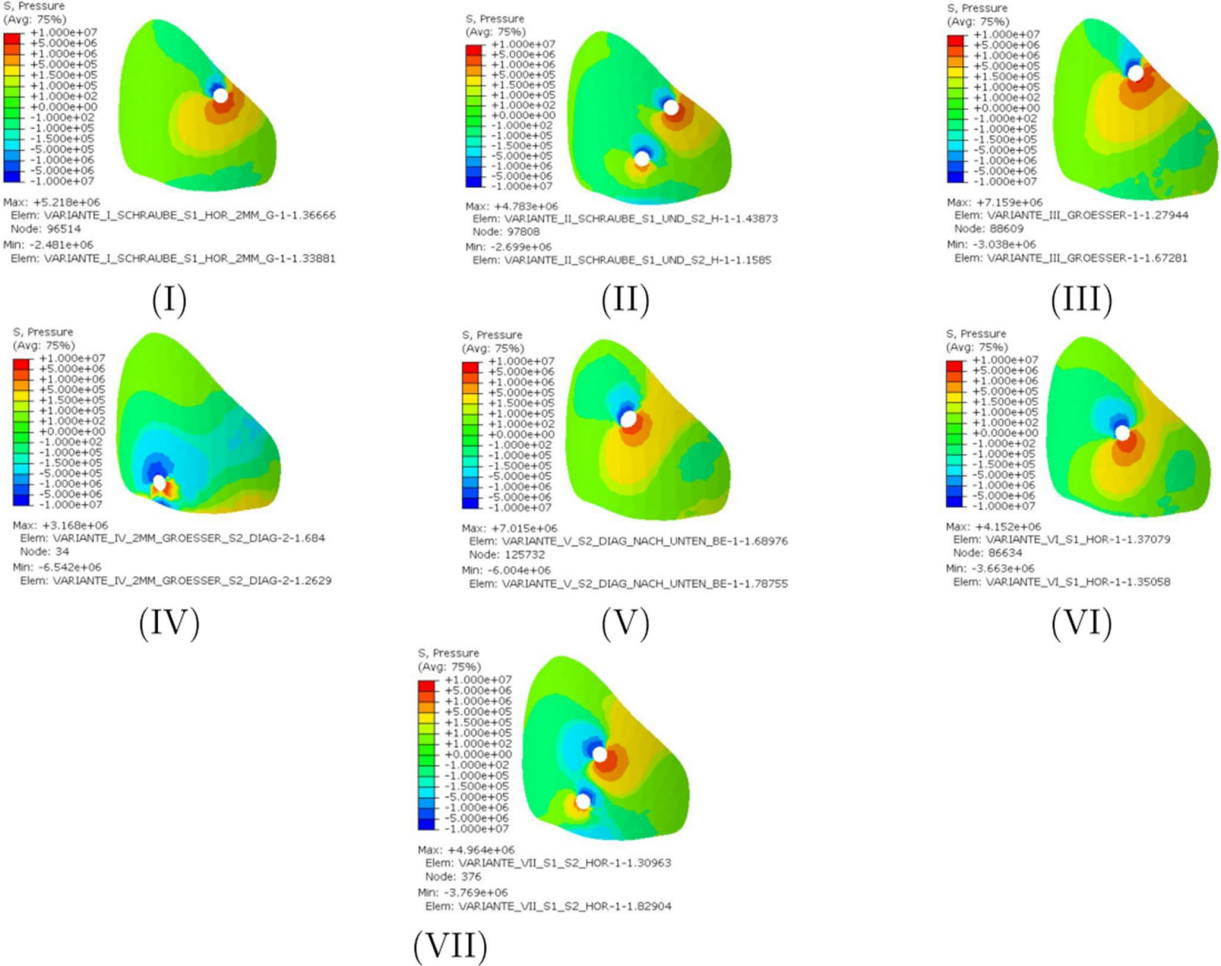
Hydrostatic pressure on the left gap surface

**Fig. 7:**
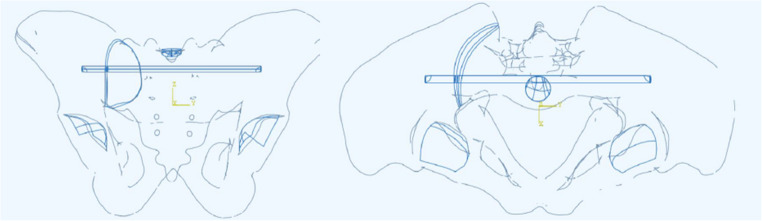
Variant I

**Fig. 8:**
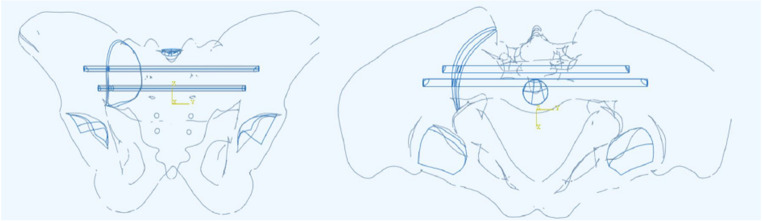
Variant II

**Fig. 9:**
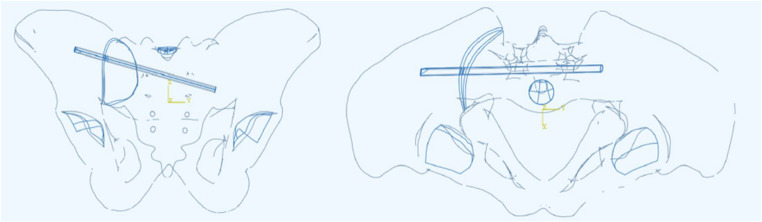
Variant III

**Fig. 10:**
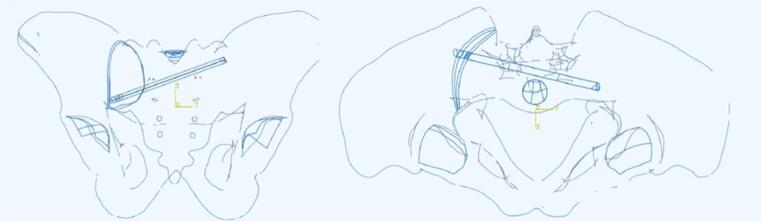
Variant IV

**Fig. 11:**
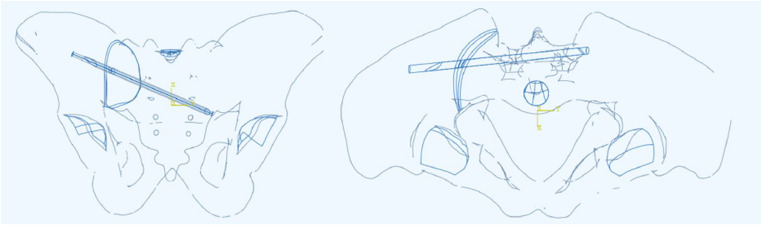
Variant V

**Fig. 12:**
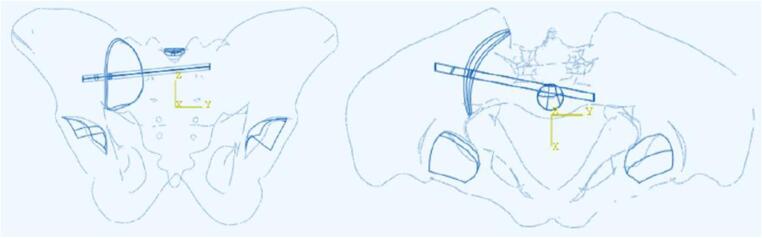
Variant VI

**Fig. 13:**
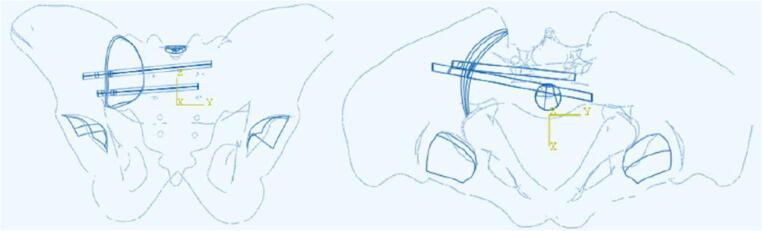
Variant VII

Figure 6 illustrates the hydrostatic pressure on the left gap surface. When comparing stresses, the sign convention is of crucial importance. Positive signs are used for compressive stresses, negative signs for tensile stresses. The stresses in the range from − 0.15 MPa to 0.15 MPa are represented by the greenish areas and occur in increased quantities in all variants. Furthermore, increased stress values can be observed in the areas of the screw entry holes. With regard to the variants examined, it can be noted that, with the exception of the Variant IV, there is a larger area subject to compressive stress with stresses above 0.15 MPa. In Variant IV, an increased area subject to tensile stress can be observed near the screw. The highest compressive stress is achieved with the third screw position and amounts to 7.16 MPa, while the lowest compressive stress can be attributed to Variant IV (3.17 MPa). However, this variant experiences the highest tensile stress at 6.54 MPa. The lowest tensile stresses occur when using a transsacral screw through the first sacral vertebral body (Variant I; 2.48 MPa).

## Discussion

With percutaneous treatment as the main stay of posterior pelvic ring stabilization, especially in geriatric patients, utilization of new screw pathways can lead to improved stability. Furthermore, navigation-assisted visualization of alternative screw corridors may expand surgical options for patients with dysplastic or osteoporotic bone quality where standard transsacral fixation is unfeasible. The objective of this finite element (FE) analysis was to investigate the feasibility of novel screw trajectories in the posterior pelvic ring made possible through 3D navigated placement, and to assess their biomechanical stability relative to conventional screw configurations. The most important findings of the study were the following:

1. Three innovative screw configurations (Variant III: oblique sacral screw through S1 pedicle to the contralateral S2 sacral ala, Variant IV: oblique sacral screw through S2 pedicle to the contralateral sacral ala at the S1 level, Variant V: oblique sacral screw through S1 pedicle to the contralateral S3 sacral cortical margin) were introduced, modeled in silico and found to be anatomically feasible.
2. Variant III achieved favorable compressive force application with reduced shear stresses at the dislocation site in comparison to the other screw position variants.
3. Variant IV demonstrated the highest overall peak stress and maximum tensile stress.

The anatomical feasibility of the proposed innovative screw pathways, which capitalize on bone corridors that are inaccessible under traditional fluoroscopic guidance, suggests navigation-assisted osteosynthesis that may broaden the surgical options for complex pelvic fractures. Conventional fluoroscopy has been established for screw placement for decades. Its widespread availability and lower costs make it the gold standard for many hospitals today, especially those with limited resources. The equipment required for fluoroscopy is less expensive to purchase and maintain than 3D navigation systems, making it a cost-effective choice for most healthcare settings [17–19]. Additionally, conventional fluoroscopy is a well-established technique with which surgeons are highly familiar, leading to faster setup times and efficient procedures [20]. On the other side, 3D navigation can facilitate reduced radiation exposure through shorter fluoroscopy time, and potentially lower reoperation rates [3, 21]. The visualization of the available osseous screw pathways from multiple angles is enabling a comprehensive understanding of the patient specific pelvic anatomy. This dynamic and continuous visual feedback is particularly valuable in complex cases where conventional imaging might not provide sufficient clarity. As a result, surgeons can confidently navigate narrow pathways around vital structures reducing the likelihood of operative errors and potentially exploit alternative biomechanically advantageous screw pathways. Before new screw corridors may be exploited, their feasibility and biomechanical properties need to be tested in silico through appropriate FE analyses.

In our study, FE simulations showed distinct differences in stress patterns across different screw corridors. Specifically, Variant III achieved favorable compressive force application with reduced shear stresses at the gap site. Hydrostatic pressure analysis on the gap surface revealed a compressive stress of up to 7.16 MPa, suggesting enhanced physiological load transfer with favorable compression-to-tension ratios. Variant IV may offer maximal mechanical compression, albeit with higher stress peaks, as it demonstrated the highest overall peak stress (von Mises up to 17.80 MPa) and maximum tensile stress (up to 16.80 MPa). This may raise potential concerns about stress concentrations in bone, which may potentially impair injury consolidation in the long term.

Conventional transsacral screw trajectories (Variants I and II) had the most uniform stress distribution and the lowest peak stress (7.17 MPa in Variant II), supporting existing literature on their biomechanical efficacy. Through FE analysis, Wu et al. (2022) showed that the first sacral vertebra osseous fixation pathway is safe and effective for sacroiliac screw pathway with optimal screw length of 130 mm, while optimizing stress distribution and minimizing displacement [22]. Lodde et al. (2025), also through FE analysis, suggested that fixation of the posterior pelvic ring with bilateral sacroiliac screws or a transsacral screw of unilateral fragility fractures of the pelvis is superior compared to the use of a single unilateral iliosacral screw [23]. Further, Zhao et al. (2012) underscored the biomechanical advantage of combined S1–S2 sacroiliac screw fixation in unstable pelvic ring injuries [24]; and Wu et al. (2023) supported combined oblique S1 and transiliac–transsacral S2 fixation in dysplastic anatomies [14].

Similar observations regarding the compressive stresses, which run along a circular or loop shape along the linea terminalis and the iliac crest are known from the literature for the healthy pelvis [25, 26]. In addition, a numerical comparison of the maximum stresses shows that they occur in the following order: IV; III; V; VI; I; VII; II. This makes it clear that the new screw oblique screw positions achieve comparable or, in some cases, lower maximum stresses. By positioning long sacral screws obliquely, the forces transmitted through the sacrum may be more efficiently distributed across a larger surface area, reducing the concentration of stress at any given point. This could lead to a lower risk of screw loosening, breakage, and or secondary displacement, particularly in regions of the sacrum that are subjected to high shear forces, like the sacral ala. The utilization of such screw pathways may reduce the mechanical load on both the screw and surrounding bone, enhancing the overall stability of the fixation.

## Limitations

Despite the insights provided by this study, limitations must be considered regarding the FE simulation of bone screws in the dislocated pelvis. First, the dislocation geometry and gap characteristics were idealized and may not fully reflect the complex biomechanical environment of actual dislocation patterns. Second, time-dependent changes in the dislocation zone through healing—such as callus formation, varying stiffness, and remodeling— were not simulated. Third, while the model applied physiological loading conditions, these were generalized and may not accurately represent patient-specific force distributions, particularly in the presence of a dislocation where load transfer through the pelvis is altered. Additionally, factors such as muscle forces, body weight variations, and asymmetrical loading during gait or rehabilitation were not included. Fourth, the bone and gap surfaces were modeled with simplified contact conditions, without fully capturing micro-motions, interfragmentary strain, or frictional effects that occur in vivo. Lastly, material properties of bone were assumed to be homogeneous, isotropic, and linear elastic, without accounting for local variations in bone quality due to age, sex, or disease (e.g., osteoporosis).

## Conclusion

In summary, our FE analysis suggests that novel navigation-assisted screw configurations are anatomically feasible and may offer biomechanical advantages over conventional iliosacral or transsacral screws in selected cases. Further, unilateral screw placement should be practiced with caution in the osteopenic sacrum as it may increase the fracture risk of the contralateral sacral side through increased stress concentration. Nevertheless, further validation is essential before integrating these pathways into routine surgical practice. Future experimental and clinical studies should verify the safety and efficacy of these novel screw trajectories under realistic physiological loading.

## Data Availability

The data collected will be stored securely at our institute for 10 years. During this period, they are still available upon request. After 10 years, the data will be deleted, however, all the datasets analyzed or generated during this study will be available from the corresponding author upon reasonable request.
